# The Effect of Pre-milking Stimulation on Teat Morphological Parameters and Milk Traits in the Italian Water Buffalo

**DOI:** 10.3389/fvets.2020.572422

**Published:** 2020-12-08

**Authors:** Angela Costa, Massimo De Marchi, Giulio Visentin, Maria Concetta Campagna, Antonio Borghese, Carlo Boselli

**Affiliations:** ^1^Department of Agronomy, Food, Natural Resources, Animals and Environment (DAFNAE), University of Padova, Legnaro, Italy; ^2^Department of Veterinary Medical Sciences (DIMEVET), University of Bologna, Bologna, Italy; ^3^Experimental Zooprophylactic Institute Lazio and Toscana “Mariano Aleandri”, Rome, Italy; ^4^International Buffalo Federation, Rome, Italy

**Keywords:** teat morphology, manual stimulation, oxytocin, dairy buffalo, udder health

## Abstract

Water buffaloes (*Bubalus bubalis*) are very sensitive to environmental stimulus before and during milking, and this explains why disrupted milk ejections due to blood oxytocin level instability are frequent in this species. According to the literature, the manual stimulation (MS) of teats before milking promotes oxytocin release and allows milk ejection to start within 2–3 min. However, the pre-milking stimulation of teats is not always part of the milking routine in Italian buffalo farms; moreover, buffaloes with unstable milk let-down are sometimes treated with exogenous oxytocin (OX). Different types of pre-milking stimulation can impact differently on the mammary gland epithelium and structures and, therefore, on milk yield. In this study, we observed the changes in teat morphological traits before and after the application of three types of stimuli, i.e., no manual stimulation (NS), MS, and OX, in buffaloes reared in an Italian dairy farm. In particular, measurements were available for 23 and 21 buffaloes for front and rear teats, respectively. Subsequently, the effect of the pre-milking stimulation type was estimated on teat morphological characteristics and on milk traits recorded after the application of stimuli. The results showed that the teat canal length was shorter (*P* < 0.05) after stimulation in the case of MS and OX compared to NS. Cistern diameter was overall greater for MS and lower for OX. On the contrary, teat wall thickness was greater and lower for OX and MS, respectively. Milk yield and quality (fat, protein, and somatic cell score) were similar across the three types of pre-milking stimulation. In perspective, the impact of these types of pre-milking stimulation should be evaluated on a large scale, and the focus might be put on mammary gland epithelium integrity, mastitis incidence, and other udder health indicators in milk, e.g., electrical conductivity, differential somatic cell count, lactose content, and sodium and chloride concentration.

## Introduction

Pre-milking udder stimulation is recommended in dairy species to promote oxytocin release in the bloodstream, reach optimal milk removal and ejection, and thus limit the stress of mammary gland. Literature shows that stimulation of teats by milker's hands seems particularly important to activate the secretory functions (1) in all dairy species. Differently from cattle, buffaloes are very sensitive to environmental stimulus before and during milking, and therefore disrupted milk ejections due to blood oxytocin level instability are more frequent (2, 3). The blood basal level of oxytocin is 4.8–6.7 ng/L in buffalo, but the concentration may reach 90 ng/L during manual stimulation, with an average of 30 ng/L during milking (2, 3). About 1–2 min of manual stimulation is generally considered appropriate in buffalo and allows milk ejection to start within 2–3 min (1, 4–6). However, in Italy, this procedure is not always performed due to the scarce knowledge of milking personnel and frequent suboptimal milking management (7–9). Considering that complete milk ejection can avoid the apoptosis of mammary gland epithelial cells and is in favor of herd productivity, buffaloes with unstable oxytocin level are sometimes treated with 20 IU of exogenous oxytocin (4). Around 10 min after the injection, the milk starts to flow. This practice is more frequent in primiparous due to higher sensitivity to the environment and greater incidence of milk let-down issues compared to multiparous; generally, around 15–20% of buffaloes in commercial farms are treated (9). Among the disadvantages related to the oxytocin use in buffalo, the risk of drug addiction and progressive resistance to oxytocin stands out; in fact, repeated administrations of this hormone may cause lack of response to normal manual stimulation in the medium to the long term (2, 3, 6).

Mammary gland epithelium and structures may face up changes after pre-milking stimulation. The type of pre-milking stimulation (e.g., none, manual, or oxytocin injection) could affect such morphological and physiological changes to a different extent (1–4). In this study, teat morphological traits were recorded before and after the application of stimuli in water buffaloes (*Bubalus bubalis*) farmed in Italy, and data on milk yield and quality were available in order to estimate the effect of the pre-milking stimulation type.

## Materials and Methods

### Design of the Study

This study was carried out in a commercial Mediterranean water buffalo herd located in the Latium region (Central Italy) from April 2013 to May 2014. Only clinically healthy buffaloes (*n* = 38), with days in milk (DIM) from 6 to 340 and from first to seventh parity were used for this experiment. Buffaloes from fourth parity onwards were grouped. The animals were milked in a herringbone (2 × 5) milking parlor (DeLaval, Tumba, Sweden). The pulsator rate was 60 cycles/min, and the pulsator ratio was 60%; during the experimental milking, the vacuum level was set at 45 kPa. The use of automatic detachment of the milking cluster was activated by the milker after the beginning of alveolar milk emission.

### Treatment

There were three pre-milking stimulation types: no manual stimulation (NS), from 1 to 2 min of manual stimulation (MS), and intramuscular injection of 20 IU of exogenous oxytocin (ipofamina 10 IU/ml, TREI—Industria Italiana Integratori S.p.a, Reggio Emilia, Italy) without manual stimulation (OX). During the study, the animals were randomly assigned to each individual treatment at each milking. All pre-milking stimulation types included the cleaning of teats with a wet disposable cotton towel.

### Teat Morphological Traits

**Figure 1:**
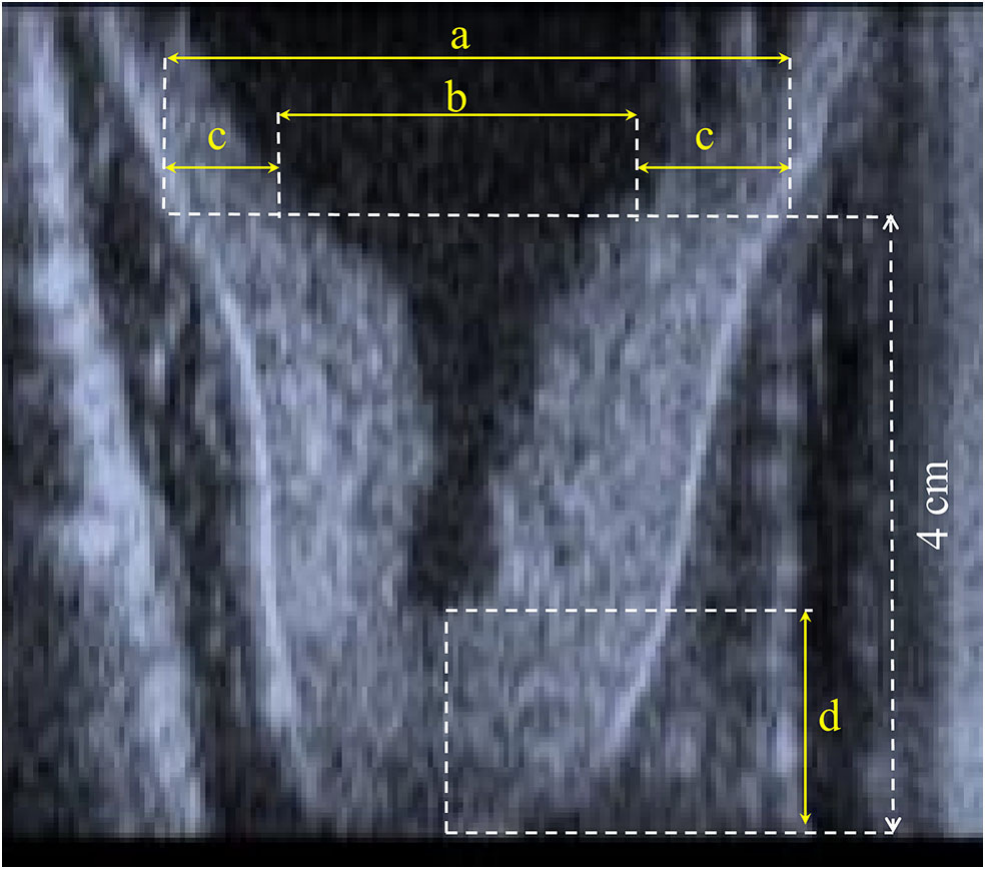
Representation of **(a)** teat diameter, **(b)** diameter of cistern, (**c**) left and right wall thickness, and **(d)** canal length.

During the whole trial, the same trained operator, a veterinarian, performed the echography to assess the teat morphological measurements before and after treatment in the same animal. Longitudinal cross-section b-mode ultrasound images (Honda HS 101V; Honda Electronics, Japan, equipped with a 5-MHz linear-array transducer) of the right front and the right rear teats were available (Figure 1) to derive the canal length (CL) and, at 4 cm above the teat tip, the teat diameter (TD), the left and the right wall thickness, and the cistern diameter (CD). The total wall thickness (WT) was calculated as the average of the left and the right wall (Figure 1). Such traits are conventionally used to study the anatomical characteristics of udder and milkability in dairy species (1–4). The images were recorded with a 5-MHz probe; the teat was put in a cup of hand-warm water, and the ultrasound probe was applied to the outside of the cup by using ultrasound gel (10, 11).

### Milk Recording Apparatus

Milk yield (kg) per quarter from the beginning to the end of mechanical milking was recorded during the milking sessions using two Lactocorder® milkmeters (WMB, Balgach, Switzerland). Each milkmeter was vertically attached to every milking place and inserted between the short milk tube and the claw. The measuring chamber consisted of one transmitting electrode and 60 vertically arranged electrodes. The production in each quarter was recorded, and a milk sample (50 ml) representative of the whole milking quarter was collected in tubes (12).

### Milk Composition

The milk samples were refrigerated (4°C) and analyzed within 24–36 h from collection at the Experimental Zooprophylactic Institute of Lazio and Tuscany “Mariano Aleandri” (Rome, Italy), the national reference laboratory for dairy product quality in Central Italy. Contents (%) of fat and protein were infrared-predicted from milk spectra using a MilkoScan FT6000 (Foss Analytics, Hillerød, Denmark) calibrated with appropriate buffalo standards, while somatic cell count (SCC, cells/ml) was determined through a flow cytometry technique with a Fossomatic FC (Foss Analytics, Hillerød, Denmark). To achieve a normal distribution of SCC data, the somatic cell score (SCS, units) was calculated as: SCS = 3 + log_2_ (SCC/100,000) (13).

### Statistical Analyses

The traits of the front and the rear teats were analyzed separately, and at least three measurements (milkings) per animal were guaranteed during the whole experimental trial. After this restriction, 138 and 129 measurements were available for the front (23 animals) and the rear (21 animals) teats, respectively. In particular, for the front teats, 44, 64, and 30 observations were available for NS, MS, and OX, while for the rear teats, 35, 63, and 21 observations were available for NS, MS, and OX. Within each pre-milking stimulation type, a *t*-test with Bonferroni–Holm step-down correction was used in SAS software, v. 9.4 (SAS Institute Inc., Cary, NC, USA) to test the difference between the mean of the trait (CL, TD, WT, or CD of either front or rear teats) recorded before and after the pre-milking stimulation (NS, MS, or OX).

Pearson correlations and their significance were obtained in the same software and were calculated between teat morphological traits separately for the front and the rear teats.

Subsequently, an analysis of variance was used to estimate the effect of the pre-milking stimulation types (NS, MS, or OX) on the teat morphological traits recorded after the pre-milking stimulation and on milk yield and composition (fat and protein content and SCS). The following mixed model was imputed in SAS software, v. 9.4 (SAS Institute Inc., Cary, NC, USA):

$$\begin{array}{l} y_{ijklm}=\mu +P_{i}+D_{j}+S_{k}+{\left(P\times D\right)}_{ij}+{\left(P\;\times \;S\right)}_{ik}+{\left(D\times S\right)}_{jk}\\ \;+B_{l}+\;e_{ijklm}, \end{array}$$

where *y*_ijklm_ is the trait recorded (morphological or milk traits) either in the front or the rear teats, μ is the overall intercept of the model, *P*_*i*_ is the fixed effect of the *i*^th^ parity of the cow (*i* = 1–4, with class 4 including parities 4–7), *D*_*j*_ is the fixed effect of the *j*^th^ class of DIM of the cow (*j* = 1–4, with DIM in class 1, 2, 3, and 4, respectively, ≤60, 61–100, 101–150, and ≥151), *S*_*k*_ is the fixed effect of the *k*^th^ pre-milking stimulation type (NS, MS, or OX), (*P* × *D*)_*ij*_ is the fixed interaction effect between parity and DIM class, (*P* × *S*)_*ik*_ is the fixed interaction effect between parity and pre-milking stimulation type, (*D* × *S*)_*jk*_ is the fixed interaction effect between DIM class and pre-milking stimulation type, *B*_*l*_ is the random effect of the *l*^th^ buffalo (three or more measurements per buffalo), and *e*_*ijklm*_ is the random residual. A multiple comparison of least square means (LSM) for the fixed effects was performed using Bonferroni's test (*P* < 0.05).

## Results

### Before vs. After Pre-milking Stimulation

The descriptive statistics of the teat morphological traits are shown in Table 1. During both before and after the pre-milking stimulation, CL was longer in the front than in the rear teats. On the other hand, the opposite was observed for TD and CD. The mean of WT before and after pre-milking stimulation was similar in the front and the rear teats. The coefficient of variation was, in general, the greatest for CD (>47.09%) and the lowest (<15.05%) for TD.

**Table 1:** Mean, coefficient of variation (CV), and range of front (*n* = 138) and rear (*n* = 129) teat measurements of morphological traits before and after pre-milking stimulation.

**Trait**	**Front^a^**			**Rear^b^**		
	**Mean**	**CV, %**	**Range**	**Mean**	**CV, %**	**Range**
**Canal length, cm**						
Before	2.548	17.91	2.20	2.401	20.27	2.40
After	2.256	24.62	2.70	2.059	28.81	3.60
**Teat diameter, cm**						
Before	3.137	14.76	2.80	3.193	15.05	2.60
After	3.134	14.84	2.60	3.217	14.59	2.50
**Wall thickness, cm**						
Before	0.920	27.18	1.20	0.925	25.11	1.05
After	0.891	30.76	1.20	0.896	29.33	1.15
**Cistern diameter, cm**						
Before	1.297	48.92	2.90	1.343	47.27	3.00
After	1.352	49.27	3.10	1.426	47.09	3.20

a *A total of 44, 64, and 30 observations were available for NS, MS, and OX*.

b *A total of 35, 63, and 21 observations were available for NS, MS, and OX*.

The correlations of the morphological traits of the front teats mirrored those of the rear teats (Table 2). Overall, the measures of the same trait before and after pre-milking stimulation were strongly correlated. For example, CD recorded before and after the pre-milking stimulation had a correlation equal to 0.98 and 0.96 for the front and the rear teats, respectively (Table 2). Negative associations were observed between CD and CL and between CD and WT, while the correlations between CL and TD and between WT and TD were not significant in both the front and the rear teats. Finally, the correlations observed between WD and CL were moderate.

**Table 2:** Correlations^a^ between morphological traits before and after pre-milking stimulation in the front and the rear teats.

**Trait**	**CL_B**,	**CL_A**,	**TD_B**,	**TD_A**,	**WT_B**,	**WT_A**,	**CD_B**,
	**cm**	**cm**	**cm**	**cm**	**cm**	**cm**	**cm**
**Front**							
CL_A, cm	0.57						
TD_B, cm	0.11^ns^	−0.02^ns^					
TD_A, cm	0.10^ns^	−0.07^ns^	0.94				
WT_B, cm	0.37	0.43	0.07^ns^	0.05^ns^			
WT_A, cm	0.29	0.44	0.06^ns^	0.08^ns^	0.96		
CD_B, cm	−0.22	−0.35	0.61	0.57	−0.74	−0.75	
CD_A, cm	−0.17^*^	−0.41	0.56	0.59	−0.75	−0.76	0.98
**Rear**							
CL_A, cm	0.70						
TD_B, cm	0.08^ns^	−0.01^ns^					
TD_A, cm	0.05^ns^	−0.11^ns^	0.91				
WT_B, cm	0.28	0.35	0.04^ns^	−0.05^ns^			
WT_A, cm	0.21	0.34	0.03^ns^	−0.01^ns^	0.95		
CD_B, cm	−0.14^ns^	−0.26	0.68	0.68	−0.70	−0.68	
CD_A, cm	−0.13^ns^	−0.33	0.57	0.66	−0.75	−0.76	0.96

a* *0.5 ≤ P < 0.10*;

ns *P ≥ 0.10*.

*CL_B, canal length before; CL_A, canal length after; TD_B, teat diameter before; TD_A, teat diameter after; WT_B, wall thickness before; WT_A, wall thickness after; CD_B, cistern diameter before; CD_A, cistern diameter after*.

The means for the trait before and after the pre-milking stimulation and the significance of *t*-test are shown in Table 3. In both the front and the rear teats, MS and OX significantly affected CL. In particular, CL was generally shorter after the pre-milking stimulation. However, the differences observed in other traits were not significant at *P* < 0.05.

**Table 3:** Means and SD of teat morphological traits before and after pre-milking stimulation and significance of *t*-test.

**Teats**	**Mean before**	**SD before**	**Mean after**	**SD after**	***P***
**Front^a^**					
No stimulation (NS)					
Cistern diameter, cm	1.302	0.660	1.312	0.661	ns
Teat diameter, cm	3.119	0.404	3.110	0.415	ns
Wall thickness, cm	0.908	0.254	0.899	0.258	ns
Canal length, cm	2.442	0.455	2.352	0.453	ns
Manual stimulation (MS)					
Cistern diameter, cm	1.413	0.643	1.503	0.668	ns
Teat diameter, cm	3.131	0.509	3.125	0.500	ns
Wall thickness, cm	0.859	0.228	0.811	0.258	ns
Canal length, cm	2.606	0.456	2.181	0.587	<0.001
Oxytocin injection (OX)					
Cistern diameter, cm	1.032	0.503	1.086	0.574	ns
Teat diameter, cm	3.179	0.452	3.189	0.458	ns
Wall thickness, cm	1.073	0.236	1.052	0.260	ns
Canal length, cm	2.580	0.448	2.278	0.595	0.016
**Rear^b^**					
No stimulation					
Cistern diameter, cm	1.344	0.600	1.344	0.599	ns
Teat diameter, cm	3.227	0.372	3.194	0.389	ns
Wall thickness, cm	0.943	0.237	0.925	0.258	ns
Canal length, cm	2.294	0.542	2.174	0.493	ns
Manual stimulation					
Cistern diameter, cm	1.473	0.659	1.609	0.697	ns
Teat diameter, cm	3.125	0.502	3.255	0.514	ns
Wall thickness, cm	0.871	0.221	0.823	0.256	ns
Canal length, cm	2.452	0.417	1.984	0.599	<0.001
Oxytocin injection					
Cistern diameter, cm	1.064	0.546	1.448	0.597	ns
Teat diameter, cm	3.107	0.547	3.166	0.469	ns
Wall thickness, cm	1.021	0.225	1.010	0.240	ns
Canal length, cm	2.419	0.546	2.086	0.673	0.036

*ns, not significant at P ≥ 0.05*.

a *A total of 44, 64, and 30 observations were available for NS, MS, and OX*.

b *A total of 35, 63, and 21 observations were available for NS, MS, and OX*.

### Comparison of the Types of Pre-milking Stimulation

**Figure 2:**
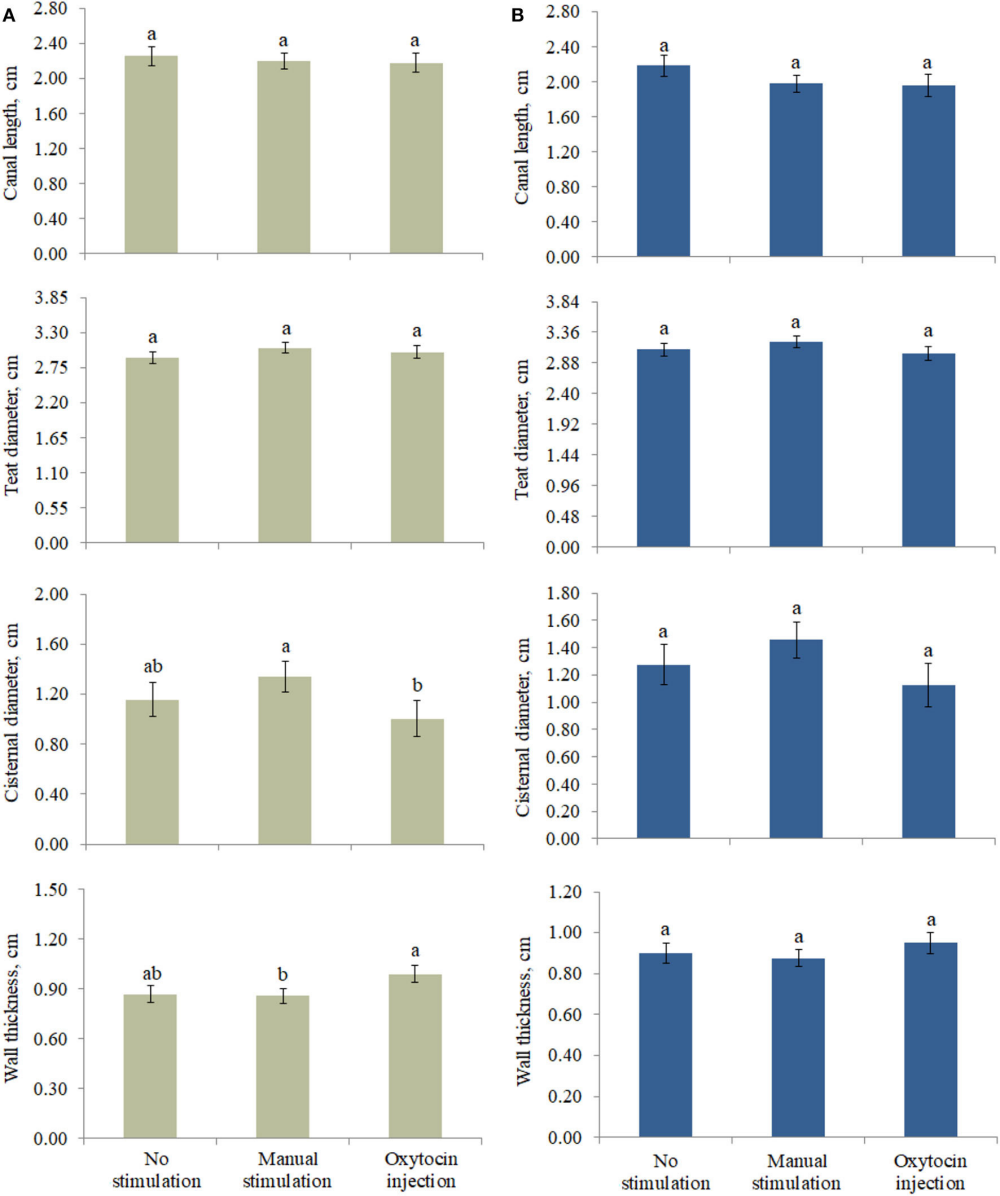
Least square means of **(A)** front and **(B)** rear teat morphological traits for the fixed effect of pre-milking stimulation. Different letters indicate statistical significance (*P* < 0.05), and bars indicate the standard errors of estimates (0.043–0.161). For no stimulation, manual stimulation, and oxytocin injection, the number of observations was equal to 44, 64, and 30 (front teats, **A**) and 35, 63, and 21 (rear teats, **B**), respectively.

The effect of the type of pre-milking stimulation was significant for CD and WT of the front teats (Figure 2). In particular, the greatest CD and the lowest WT were estimated in the teats that were manually stimulated (Figure 2). On the other hand, the animals that were treated with oxytocin had front teats with the lowest CD and the greatest WT. Intermediate LSM were estimated for the NS teats.

At both the front and the rear levels, the quantity and the quality of milk were similar among NS, MS, and OX teats (Table 4). Despite being not significant, fat content seemed to be greater in NS (front teats) and OX (rear teats); the protein content of the milk of the front teats was the greatest in OX (4.849 %), while in the rear teats it had scarce variation among the three pre-milking stimulation types (Table 4). Milk yield was greater in the rear quarters than in the front ones in all the applied treatments, ranging from 0.883 kg (rear teats, NS group) to 1.599 kg (rear teats, OX group). Although milk yield was maximum in general for OX, the LSM of NS, MS, and OX did not differ significantly (Table 4).

**Table 4:** Least squares means and standard errors (SE) of front and rear teat milk yield and composition traits for the fixed effect of pre-milking stimulation.

**Teats**	**No stimulation (NS)**	**SE**	**Manual stimulation (MS)**	**SE**	**Oxytocin injection (OX)**	**SE**
**Front^a^**						
Milk, kg	0.883a	0.099	0.856a	0.091	0.917a	0.109
Fat, %	8.862a	0.465	8.417a	0.448	8.629a	0.623
Protein, %	4.775a	0.119	4.696a	0.116	4.849a	0.153
SCS, units	3.022a	0.320	2.199a	0.307	2.584a	0.433
**Rear^b^**						
Milk, kg	1.376a	0.129	1.366a	0.118	1.599a	0.142
Fat, %	8.942a	0.461	8.114a	0.465	9.409a	0.627
Protein, %	4.748a	0.101	4.790a	0.102	4.761a	0.136
SCS, units	3.001a	0.359	2.592a	0.342	2.877a	0.489

a *A total of 44, 64, and 30 observations were available for NS, MS, and OX*.

b *A total of 35, 63, and 21 observations were available for NS, MS, and OX*.

*Different letters within a row indicate statistical significance (P < 0.05)*.

**Figure 3:**
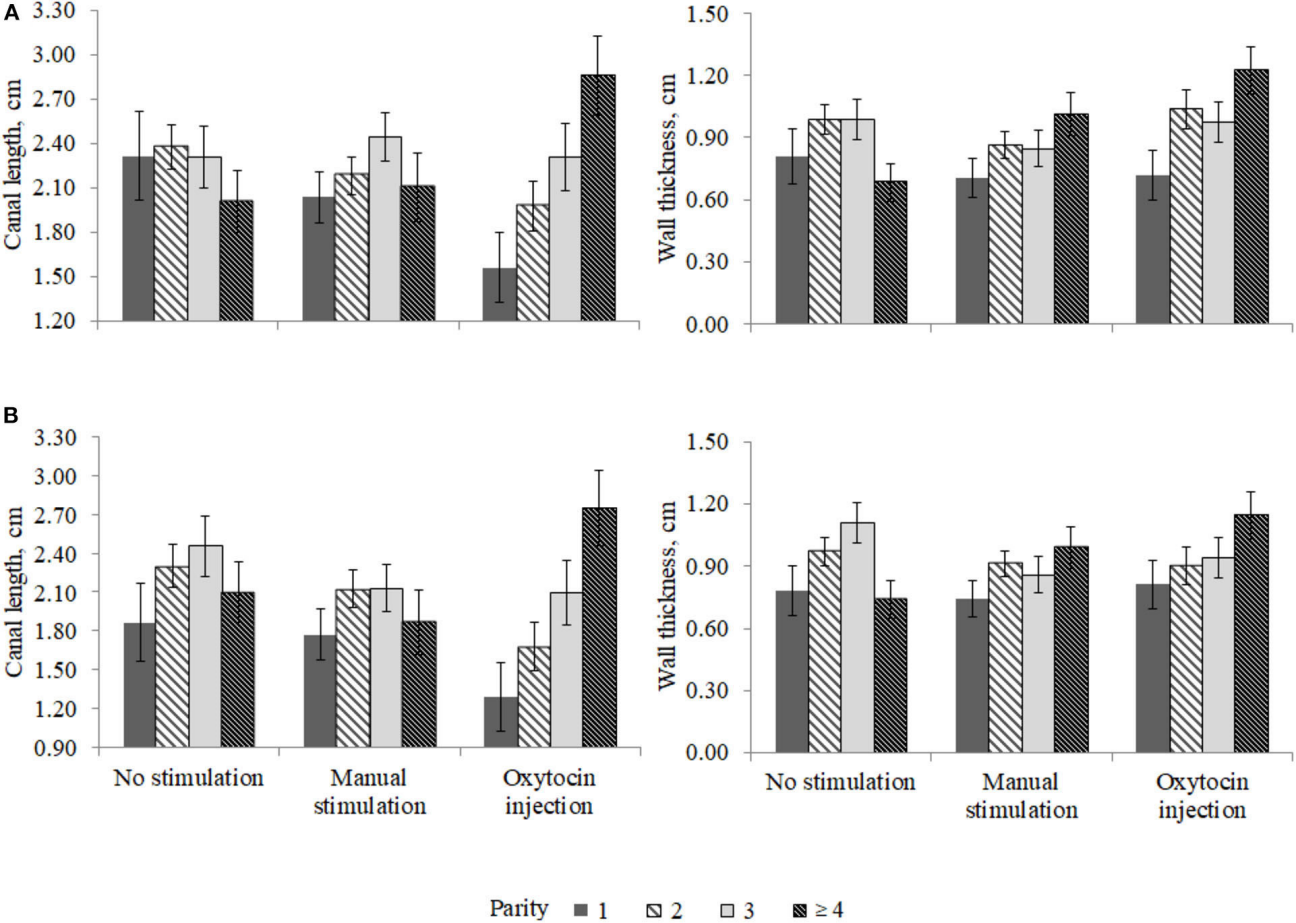
Least square means of **(A)** front and **(B)** rear teat canal length and wall thickness for the fixed effect of the interaction between parity and pre-milking stimulation.

The interaction effect between the pre-milking stimulation type and parity was relevant for the front (*P* < 0.02) and the rear (*P* < 0.08) teat WT and for the front (*P* < 0.03) and the rear (*P* < 0.03) teat CL and is depicted in Figure 3. In particular, the front teat CL of primiparous buffaloes was the lowest in OX (1.56 ± 0.24 cm) and the greatest in NS (2.32 ± 0.30 cm; Figure 3). A similar trend was observed for buffaloes in second parity (Figure 3). Third-parity buffaloes presented LSM of CL equal to 2.31 ± 0.21, 2.44 ± 0.17, and 2.31 ± 0.23 cm for NS, MS, and OX, respectively. An opposite trend was observed for LSM of buffaloes in fourth parity, i.e., the CL was the lowest in NS (2.01 ± 0.21 cm) and the greatest in OX (2.86 ± 0.27 cm). In both the front and the rear teats, the greatest CL was estimated for fourth-parity buffaloes in the OX group (Figure 3).

## Discussion

The average measurements (Table 1) before the stimuli were comparable to the findings observed in a field study conducted on 18 Mediterranean buffalo in Italy (14), where, using both the front and the rear teats, the authors found average CL, TD, WT, and CD equal to 2.55, 3.27, 0.97, and 1.31 cm, respectively. The mean CL was similar in the front (2.63) and the rear (2.78) positions (14). The average CL (3.10 cm) recorded in 24 Murrah buffaloes was slightly greater compared with the findings observed in the present study (15); in the same study, the authors observed different CL between the rear (3.7 cm, on average) and the front teats (3.0 cm, on average), but no differences in CL among parities and stages of lactation. In this study, both TD and CD were larger in the rear than in the front teats; this was in agreement with the studies on Murrah buffaloes (15, 16). In fact, a greater cisternal area in the rear (11.84 cm ^2^) than in the front (9.57 cm^2^) quarters was observed. In Mediterranean Italian buffalo (14), the TD was significantly greater in the rear than in the front quarters (6).

The correlations between TD and CL were not significant, which is likely due to the limited sample size and to a weak to null dependency between the features. Within trait, the correlations suggested a strong (>0.9) dependency between the measures recorded before and after the pre-milking stimulation, except for CL (Table 2). In fact, the correlation of CL before and after the application of stimuli was 0.57 in the front teats and 0.70 in the rear teats, suggesting that the type of stimulation may have a different effect on the change of CL. This is also supported by the results of the *t*-test (Table 3). In fact, among all traits, only CL was significantly different before and after the stimuli in the case of OX and, more particularly, of MS (*P* < 0.001; Table 3). This finding was in line with a recent study conducted on Anatolian buffaloes (11), where significant reductions in CL in the fore and the rear quarters after 3 and 6 min of manual pre-stimulation were observed. In general, according to the literature, a reduction in CL was observed after manual stimulation or the administration of oxytocin (1, 4, 11).

In a study based on 38 dairy cows (17), CL and WT were moderately correlated at the quarter level; the correlation between CL and TD was weak instead.

The animals not stimulated before milking did not present changes in (front and rear) teat morphological traits. With regard to the front teats, the pre-milking stimulation type significantly affected CD and WT, but it is worth to highlight that some differences observed in this study (Figure 2) were not significant, which is likely due to the high standard error of estimates. For the same reason, the milk traits estimated for the three pre-milking stimulation types did not differ (Table 4); however, the LSM of milk yield was maximum (0.917 ± 0.109 and 1.599 ± 0.142 kg for the front and the rear teats, respectively) in OX, supporting the idea that exogenous oxytocin promotes complete milk ejection (4, 6). The rear quarters in buffaloes are slightly larger than the front ones and contain more milk. The approximate ratio is 60:40 (rear/front) as for cattle (16, 17). In support of this, the ratio in our study ranged from 61:39 (NS and MS) to 64:36 (OX).

In a field study (6), the difference in milk yield at the udder level between buffaloes treated and not treated with oxytocin was 10–12%; in the present study, the milk yield of the rear teats in buffaloes treated with oxytocin was 13.95% greater than in animals not treated and 14.57% greater than in the manually stimulated animals (Table 4). In the front teats, milk yield was 3.71–6.65% higher if oxytocin was used (Table 4). Although not significant, SCS seemed to be worse in NS and the best in MS in both the front and the rear teats. According to the literature (18), the use of oxytocin does not change milk SCS at milking, but no studies have evaluated the effect of prolonged OX on udder health indicators and/or on mastitis incidence (18).

The differences in teat morphological traits (CL and WT) observed among parities (Figure 3) may indicates that oxytocin acts differently in the parities considered. Nevertheless, it should be highlighted that the present study was based on a small dataset collected in one farm; thus, the statistical power was relatively low, and some potentially significant differences were not found.

## Conclusions

An adequate udder pre-milking stimulation is usually recommended in dairy species for complete milk removal and optimal milk ejection. In this study, three types of stimuli were investigated: NS, MS, and OX. Both MS and OX caused a reduction in the CL of both the front and the rear teats. Milk yield and quality were similar across the three types of pre-milking stimulation, while significant differences were observed in CD and WT. In particular, the manually stimulated teats showed greater CD and lower WT, while the buffaloes treated with oxytocin had lower CD and greater WT; this was also supported by the correlations estimated in this study. In fact, after the application of stimuli, CD and WT were negatively correlated in both the front (−0.76) and the rear (−0.76) teats. The results suggest that OX may lead to complete milk removal in buffaloes, with apparently no impact on milk quality. Considering that only one farm was involved in this study, these findings may be considered and interpreted with caution. Future experimental trials may be designed in order to include more farms and should evaluate the effect of pre-milking stimulation type on mammary gland epithelial integrity, clinical and subclinical mastitis incidence, and milk indicators of udder health and alveolar permeability, like milk electrical conductivity, differential somatic cell count, lactose content, and sodium and chloride concentration.

## Data Availability Statement

The raw data supporting the results and the conclusions of this article will be made available by the authors, without undue reservation.

## Ethics Statement

Ethical approval was not required for the animal study as per institutional guidelines/local legislation. The owner of the animals used in the study provided written informed consent.

## Author Contributions

This work was conceived and designed by all the authors. AB and CB were in charge of animal handling and data collection. AC and GV performed the analyses and interpreted the results. The manuscript was mainly written by AC, CB, and AB. MD and MC coordinated the activities and revised the manuscript. GV revised the manuscript and handled the revision process. All the authors contributed to read and approved the final version submitted.

## Conflict of Interest

The authors declare that the research was conducted in the absence of any commercial or financial relationships that could be construed as a potential conflict of interest.

## References

[1] Ambord S Stoffel MH Bruckmaier RM. Teat anatomy affects requirements for udder preparation in Mediterranean buffaloes. J Dairy Res. (2010) 77:468–73. 10.1017/S0022029910000518 20822559

[2] Thomas CS Borghese A D'Elisi MG Physiology of milk ejection. In: Rasmussen M Thomas CS Borghese A editors. Milking Management of Dairy Buffaloes. Brussels: Bulletin of the International Dairy Federation n. 426/2008 (2008). p. 31–5.

[3] Thomas CS Bruckmaier RM Ostensson K Sjaunja KS. Effect of different milking routines on milking-related release of the hormones oxytocin, prolactin and cortisol, and on milk yield and milking performance in Murrah buffaloes. J Dairy Res. (2005) 72:10–8. 10.1017/S0022029904000457 15747726

[4] Boselli C Campagna MC Amatiste S Rosati R Borghese A Pre-stimulation effects on teat anatomy and milk flow curves in Mediterranean Italian Buffalo cows. J Anim Vet Adv. (2014) 13:912–6. 10.3923/javaa.2014.912.916

[5] Shahid MQ Abdullah M Bhatti JA Javed K Babar ME Jabbar MA Machine milking performance of Nili Ravi buffaloes on different pre-milking stimulation practices. J Anim Plant Sci. (2012) 3:284–7. Available online at: http://www.thejaps.org.pk/docs/Supplementary/03/032.pdf

[6] Neglia G Saltalamacchia F Thomas CS Rasmussen MD Milking routines. In: Rasmussen M Thomas CS Borghese A editors. Milking Management of Dairy Buffaloes. Brussels: Bulletin of the International Dairy Federation n. 426/2008 (2008). p. 69–83.

[7] Bava L Sandrucci A Tamburini A Zucali M Milk flow traits of buffalo cows in intensive farming systems. Ital J Anim Sci. (2007) 6:500–2. 10.4081/ijas.2007.1s.500

[8] Boselli C Rosati R Giangolini G Arcuri S Fagiolo A Ballico S Milk flow measurements in buffalo herds. In: Proc. of the 7^th^ World Buffalo Congress, Manila, Philippines (2004).

[9] Di Palo R Campanile G Ariota B Vecchio D Grassi C Neri D Milk flow traits in Mediterranean Italian Buffaloes. Ital J Anim Sci. (2007) 6:1319–22. 10.4081/ijas.2007.s2.1319

[10] Ambord S Thomas C S Borghese A Mazzi M Boselli C Bruckmaier RM. Teat anatomy, vacuum to open the teat canal, and fractionized milk composition in Italian buffaloes. Milchwissenschaft. (2009) 64:351–3. Available online at: https://www.cabdirect.org/cabdirect/abstract/2009328322020822559

[11] Ozenc E Bozkurt MF Yazici E Seker E Bayraktaroglu AG Ozcinar U. Teat characteristics in relation to animal temperament during milking in buffaloes, and comparison of buffalo and cow teat morphology. Reprod Domest Anim. (2020) 55:559–66. 10.1111/rda.13650 31997393

[12] Instructions Lactocorder. Available online at: http://www.lactocorder.ch/startframeset.asp?l=en (accessed March 1, 2020).

[13] Ali AKA Shook GE An optimum transformation for somatic cell concentration in milk. J Dairy Sci. (1980) 63:487–49. 10.3168/jds.S0022-0302(80)82959-6

[14] Boselli C Mazzi M Borghese A Terzano GM Giangolini G Filippetti F Milk flow curve and teat anatomy in Mediterranean Italian buffalo cows. In: Proc. of the 9^th^ World Buffalo Congress, Buenos Aires, Argentina (2010)

[15] Thomas CS Svennersten-Sjaunja K Bhosrekar MR Bruckmaier RM. Mammary cisternal size, cisternal milk and milk ejection in Murrah buffaloes. J Dairy Res. (2004) 71:162–8. 10.1017/S0022029904000081 15190943

[16] Thomas CS Borghese A Cuscunà FP. Anatomy of the buffalo udder. In: Rasmussen M Thomas CS Borghese A editors. Milking Management of Dairy Buffaloes. Brussels: Bulletin of the International Dairy Federation n. 426/2008. (2008). p. 21–7.

[17] Weiss D Weinfurtner M Bruckmaier R.M. Teat anatomy and its relationship with quarter and udder milk flow characteristics in dairy cows. J Dairy Sci. (2004) 87:3280–9. 10.3168/jds.S0022-0302(04)73464-5 15377607

[18] Bidarimath M Aggarwal A. Studies on cisternal and alveolar fractions and its composition and mammary health of Murrah buffaloes administered oxytocin. Trop Anim Health Prod. (2007) 39:433–8. 10.1007/s11250-007-9042-0 17966274

